# Children with developmental coordination disorder display atypical interhemispheric connectivity during conscious and subconscious rhythmic auditory-motor synchronization

**DOI:** 10.1038/s41598-024-69807-4

**Published:** 2024-08-28

**Authors:** Marija Pranjić, Jason Leung, Ka Lun Tam, Helene Polatajko, Timothy Welsh, Tom Chau, Michael Thaut

**Affiliations:** 1 Music and Health Research Collaboratory, Faculty of Music, University of Toronto, Toronto, Canada; 2 Holland Bloorview Kids Rehabilitation Hospital, Bloorview Research Institute, Toronto, Canada; 3 Department of Occupational Science and Occupational Therapy, Rehabilitation Sciences Institute, Faculty of Medicine, University of Toronto, Toronto, Canada; 4 Centre for Motor Control, Faculty of Kinesiology & Physical Education, University of Toronto, Toronto, Canada; 5 Institute of Biomedical Engineering, University of Toronto, Toronto, Canada; 6 Institute of Medical Science and Rehabilitation Research Institute, Faculty of Medicine, University of Toronto, Toronto, Canada

**Keywords:** Neuroscience, Psychology, Health care, Medical research

## Abstract

Children with developmental coordination disorder (DCD) display difficulties in perception-action coupling when engaging in tasks requiring predictive timing. We investigated the influence of awareness on auditory-motor adjustments to small and large rhythmic perturbations in the auditory sequence to examine whether children synchronize their movements automatically or through planning and whether those adjustments occur consciously or subconsciously. Electroencephalography (EEG) was used to assess functional connectivity patterns underlying different adjustment strategies. Thirty-two children aged 7–11 participated, including children with DCD and their typically developing (TD) peers with and without musical training. All children automatically adjusted their motor responses to small rhythmic perturbations by employing the anticipatory mode, even when those changes were consciously undetectable. Planned adjustments occurred only when children consciously detected large fluctuations (Δ 20%), which required a shift from predictive to reactive strategies. Compared to TD peers, children with DCD showed reduced interhemispheric connectivity during planned adjustments and displayed similar neural patterns regardless of task constraints. Notably, they benefited from rhythmic entrainment despite having increased variability and lower perceptual acuity. Musical training was associated with enhanced auditory-perceptual timing, reduced variability, and increased interhemispheric coherence. These insights are important for the therapeutic application of auditory/rhythm-based interventions in children with DCD.

## Introduction

Predictive processes play an essential role in perceptual-motor interactions, enabling the acquisition of accurate, smooth, and flexible movements^1^. While the link between perception and action had been largely overlooked until the 1970s^2^, more recent theoretical frameworks have focused on the interplay between the two^3^. In the context of human motor behavior, auditory rhythms have been used as a model to probe predictive timing mechanisms^4–6^ as they provide intervallic, temporal regularities that are inherently predictive, facilitating communication between auditory and motor networks even in the absence of movement^7,8^. Specifically, predictive auditory rhythms have been shown to engage the cortical and subcortical motor regions such as the premotor cortex, supplementary motor area, inferior parietal lobule, basal ganglia, and cerebellum [for reviews, see^9–12^]. Furthermore, the internal predictive processes subserving rhythmic auditory-motor entrainment are thought to occur via the dorsal auditory stream, a neural pathway that connects auditory and motor brain areas^5,6^. This coupling has been described as enactive^4^ because it enables the brain to generate predictions through active inference of perceptual inputs^13^. In other words, rather than depending on passive, bottom-up processes, perceptual contributions are thought to be constructive and interconnected with cognitive processes, resulting in reduced prediction error^14^.

Valuable insights concerning predictive timing mechanisms come from sensorimotor finger-tapping paradigms, where participants are asked to synchronize their finger taps with the auditory cue^9^. The results of studies using synchronization paradigms point to two important mechanisms supporting auditory-motor interactions. First, they show that participants begin to anticipate the incoming rhythmic stimuli, rather than simply reacting to them, by tapping slightly ahead of the beat^15^. This phenomenon is known as Negative Mean Asynchrony, and it is an indicator of predictive coding that emerges when the motor response becomes entrained to the rhythmic beat^16^. Second, synchronization paradigms show that humans can make movement adjustments in response to changing rhythmic stimuli even at levels below conscious perception^17–21^. This suggests that phase error correction—the time difference between the tap and the tone—is not constrained by the perceptual threshold^17,22,23^. In contrast, when perceptible timing perturbations are introduced, multiple conscious synchronization strategies involving the initiation and planning of motor movements may be employed, depending on task constraints^22,23^.

It is important to highlight that most of the evidence in this line of inquiry comes from research with neurotypical adults. Therefore, less is known about auditory-motor adjustment strategies in children, particularly those with motor difficulties such as developmental coordination disorder (DCD), which affects approximately 5–6% of school-aged children^24^. Research suggests that children with DCD display differences in perception-action coupling as well as atypical neural activation patterns when engaging in tasks that involve predictive timing^25,26^. A recent study found that children at risk for DCD also displayed perceptual differences in rhythmic timing in the absence of motor responses^27^. The perceptual thresholds were further corroborated by the delayed mismatch negativity latencies for duration timing and delayed P3a latencies in response to rhythm deviants. Thus, there is an indication that auditory perceptual timing may be impacted in children with developmental coordination disorder^28^ in addition to their core difficulties in motor control. To our knowledge, only one study tested synchronization abilities in children with DCD by employing a paradigm requiring motor adjustments to perceptible and subliminal (i.e., not consciously perceived) perturbations in an auditory sequence^29^. This study found no significant differences in perceptual thresholds and motor adjustments to gradual and abrupt changes in rhythmic stimuli between 6 to 11-year-old children with and without DCD. Their results suggest that the increased synchronization variability characteristic for children with DCD does not stem from poor perceptual timing abilities. However, their results should be interpreted cautiously because Roche and colleagues^29^ did not employ the adaptive threshold estimation paradigms commonly used in psychophysical research^30^. It is also plausible that some children perceived their stimulus phasing change of 12.5% on a subliminal rather than perceptible level, considering the participants were not required to report whether they perceived rhythmic fluctuations across tasks.

Stephan and colleagues^19^ described this challenge of differentiating between subconscious and conscious adaptations as a double dissociation wherein (1) motor adjustments can be produced either automatically or through conscious planning, while (2) participants may or may not be aware of their actions irrespective of whether the adjustments were produced automatically. In their study, Stephan and colleagues^19^ aimed to assess the influence of awareness on tapping adjustments using a synchronization paradigm that involved continuous time-modulated rhythmic perturbations at 3, 7, and 20% of change while measuring regional cerebral blood flow. Although none of the participants consciously perceived the fluctuations at 3% and only some perceived changes at 7%, they still employed an anticipatory mode in both conditions and produced motor adjustments automatically. The results also showed that intentional motor adaptation strategies emerged only when participants consciously detected the changes in rhythmic stimuli at 20%, corresponding with activations of distinct prefrontal areas, including the dorsolateral prefrontal cortex. Therefore, cognitive strategies begin to play a role when changes in rhythmic patterns are consciously perceived.

Cognitive differences have repeatedly been identified as an area of pronounced difficulty in children with DCD^31^, and cognitive-based strategies have proved effective in improving motor performance compared to bottom-up, process-oriented approaches^32,33^. Still, it is unclear how cognitive, perceptual, and motor processing differences may affect synchronization strategies on both subliminal and perceptible levels. Moreover, it is unknown whether extensive musical training in childhood may contribute to enhanced auditory-motor adjustments. Examining and controlling for the effects of musical training is crucial in auditory-motor research since long-term musical training has been shown to improve rhythmic timing abilities across both perceptual and motor domains^34,35^. However, studies focusing on auditory-motor skills in children with musical training are limited, and the link between musical training and DCD symptomatology remains unexplored.

The present study aimed to fill these knowledge gaps by investigating the role of awareness in auditory-motor synchronization in children with DCD and their typically developing peers with and without extensive musical training. We adopted and modified the synchronization paradigm developed by Stephan and colleagues^19^ to examine whether children with varying levels of motor and auditory-perceptual abilities adjust their movements to cosine-wave modulated rhythmic sequences automatically or through conscious planning and whether those adjustments occur consciously or subconsciously. In addition to behavioral measures, we employed electroencephalography (EEG) to examine functional connectivity patterns underlying conscious and subconscious motor adjustments. EEG was used given its high temporal resolution, safe and noninvasive nature, and strong potential to identify biomarkers in neurodevelopmental conditions. We hypothesized that all children would perceive small frequency fluctuations (3% and possibly 7%) only on a subliminal level and would make motor adjustments subconsciously. Large frequency fluctuations (20%) would be consciously recognized, and participants would start to plan their motor actions intentionally, leading to distinct functional connectivity patterns across fronto-central networks. We further anticipated that children with DCD would exhibit increased tapping variability across tasks, while musical training would contribute to enhanced perceptual recognition of rhythmic perturbations in the auditory stimuli. Finally, based on sensorimotor research in adult musicians^34,35^, we hypothesized that greater synchronization accuracy would be reflected by enhanced interhemispheric connectivity in the fronto-central networks.

## Results

### Participants

We report the behavioral data from all 32 participants and the neurophysiological data for a total of 24 (DCD *n* = 7; TD *n* = 8; TDM *n* = 9). The EEG data from two participants in the DCD group and three in the TD groups were excluded due to unsatisfactory signal quality due to motion artifacts. Signal quality was assessed using the HAPPE processing report metrics^36^ and was considered low if the percentage of good channels was below 65% or if the electrodes overlying the primary cortex were heavily contaminated. The groups did not significantly differ in age, cognitive abilities, immediate auditory attention, working memory abilities, and household income (see Table 1). Regarding ethnicity, there were more non-Caucasian participants in the musician group. As expected, motor coordination difficulties were present in the DCD group only. Caregiver questionnaires also revealed the presence of coexisting ADHD symptoms for two children in the DCD group. Regarding auditory-perceptual thresholds, the groups did not differ when detecting changes in tone durations in the absence of an underlying beat (i.e., interval-based timing) [*F*_(2,29)_ = 2.73, *p* = 0.082, η_p_^2^ = 0.16]. In contrast, there was a significant difference in the rhythm-based discrimination task [*F*_Welch(2,13.051)_ = 14.62, *p* < 0.001, η_p_^2^ = 0.42], wherein musicians (TDM) displayed significantly lower perceptual thresholds (i.e., enhanced perceptual acuity) than both the DCD [*t*_(19)_ = 4.21, *p* < 0.001,* d* = 1.85] and TD groups [*t*_(21)_ = 4.49, *p* < 0.001,* d* = 1.87], while the DCD group had the largest thresholds. Participant characteristics did not differ between the full sample and the subgroup of children who completed EEG (for details, see Table S1).

**Table 1:** Participant characteristics and the auditory-perceptual thresholds by group.

	DCD	TD	TDM	*p*-value
*N*	9	11	12	–
Age (months)	111.33 ± 17.56	107.64 ± 15.17	113.67 ± 18.64	.704
Non-Caucasian	44.4%	45.5%	56.2%	.037*
Income < $100,000	33.3%	45.5%	16.7%	.102
Laterality quotient (RH)	94.44%	100%	98.88%	.375
KBIT-2 Composite IQ	108.33 ± 20.64	115.91 ± 9.62	119.92 ± 8.82	.161
DST Forward	8.22 ± 1.40	8 ± 1.61	8.92 ± 1.68	.363
DST Backward	3.89 ± 1.97	5.36 ± 2.73	6.33 ± 2.23	.078
MABC-2 Percentile	7.67 ± 4.03	65.73 ± 16.60	59.17 ± 12.56	< .001*
DCD-Q	30.67 ± 8.85	66.64 ± 6.36	68.58 ± 5.81	< .001*
Duration discrimination	34.56 ± 5.41	35.15 ± 4.90	30.79 ± 4.31	.082
Rhythm discrimination	22.18 ± 6.43	19.19 ± 3.60	14.27 ± 1.16	< .001*

Difference tests were one-way ANOVAs (reported as M ± SD) and chi-square tests for categorical variables.

*DCD* developmental coordination disorder, *TD* typically developing children, *TDM* typically developing musicians, *Laterality Quotient (RH*) right-handedness measures by Edinburgh Handedness Inventory, *KBIT-2* The Kaufman Brief Intelligence Test, 2nd Edition, *MABC-2* The Movement Assessment Battery for Children, 2nd Edition, *DST* Digit Span Test, *DCD-Q* Developmental Coordination Disorder Parent Questionnaire.

*Indicates a significant difference between groups; *p* < .05.

### Auditory-motor synchronization adjustments

**Figure 1:**
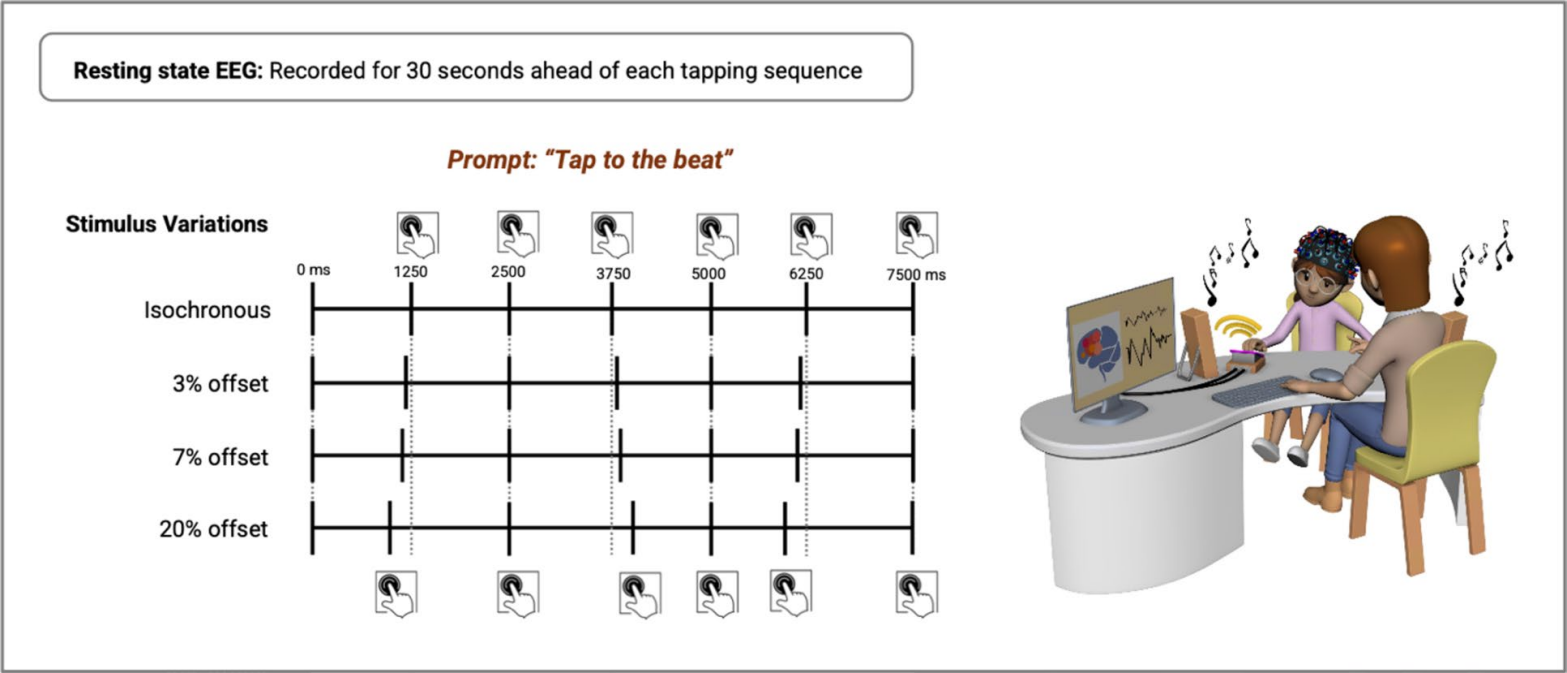
Illustration of the experimental paradigm and auditory-motor synchronization patterns (adapted from^19^). Participants were instructed to sit calmly for 30 s before each tapping sequence. Across all four tapping conditions (i.e., isochronous, 3, 7, and 20% change), children were prompted to synchronize their taps with the auditory cue as accurately as possible. Each condition was repeated twice, and each tapping sequence lasted 75 s.

As expected, synchronization error was significantly different when children were making motor adjustments to large perturbations (i.e., ∆ 20%, wherein ∆ represents the change in the stimulus) compared to conditions with smaller or no perturbations (i.e., 0, ∆ 3, ∆ 7%) [main effect of Condition; *F*_(1.652, 47.912)_ = 19.20, *p* < 0.001, η_p_^2^ = 0.40], while 0−∆ 3%, 0−∆ 7%, and ∆ 3−7% comparisons did not significantly differ (see Fig. 1A). Negative mean asynchronies suggest that children tapped slightly ahead of the beat (i.e., anticipatory mode) when synchronizing to small or no change in the auditory beat (for mean values, see Table 2). Since all positive and negative values were averaged to calculate the relative phase error for each participant, the small SE observed in the ∆ 20% tapping condition should be interpreted along with the measures of variability (i.e., SD of ITI and SD of SE). When large perturbations were present, the variability significantly increased for all three groups [SD of SE; Condition, *F*_(3, 87)_ = 131.26, *p* < 0.001, η_p_^2^ = 0.82; SD of ITI; Condition, *F*_(1.961, 56.857)_ = 17.24, *p* < 0.001, η_p_^2^ = 0.37]. Furthermore, children with DCD were significantly more variable than their TDM peers during isochronous tapping [SD of SE; Group, *F*_(2, 29)_ = 6.97, *p* < 0.003, η_p_^2^ = 0.32; *t*_(19)_ = 4.17, *p* < 0.001,* d* = 1.83], at ∆ 7% [*t*_(19)_ = 3.06, *p* = 0.006,* d* = 1.35], and ∆ 20% [*t*_(19)_ = 3.01, *p* = 0.007,* d* = 1.33] (see Fig. 1B). The effects persisted after covarying for age [Group, *F*_(2, 28)_ = 9.41, *p* < 0.001, η_p_^2^ = 0.40; Condition, *F*_(3, 84)_ = 5.49, *p* = 0.002, η_p_^2^ = 0.16]. The mean inter-tap interval remained similar across conditions, indicating that the rhythmic perturbations did not significantly affect period adjustments.

**Table 2:** Means and standard deviations of the synchronization measures for all conditions.

	DCD (*n* = 9)	TD (*n* = 11)	TDM (*n* = 12)	*p*-value
ISI (ms)	1250	1250	1250	–
ITI 0%	1252.62	1252.02	1251.30	Group = .173 Condition = .778 Group × Condition = .976
ITI Δ 3%	1248.69	1251.35	1250.38	
ITI Δ 7%	1249.19	1250.16	1251.24	
ITI Δ 20%	1250.80	1250.04	1251.97	
SD of ITI 0%	208.93	150.86	155.76	Group = .502 Condition < .001* Group × Condition = .888
SD of ITI Δ 3%	167.18	172.29	183.57	
SD of ITI Δ 7%	216.22	175.87	155.05	
SD of ITI Δ 20%	333.27	314.10	316.89	
SE 0%	−116.91	−48.72	−50.75	Group = .119 Condition < .001* Group × Condition = .109
SE Δ 3%	−111.27	−46.07	−58.49	
SE Δ 7%	−139.11	−53.43	−62.09	
SE Δ 20%	−18.13	−17.95	16.50	
SD of SE 0%	183.17	125.76	85.98	Group = .003* Condition < .001* Group × Condition = .668
SD of SE Δ 3%	181.05	149.19	104.86	
SD of SE Δ 7%	190.83	163.13	130.95	
SD of SE Δ 20%	317.24	275.51	236.92	

Negative mean values indicate that the taps preceded the auditory beat (i.e., anticipatory mode).

*DCD* developmental coordination disorder, *TD* typically developing, *TDM* typically developing musicians, *ISI* inter-stimulus interval, *ITI* inter-tap interval, *SD *standard deviation, *SE* synchronization error (relative phase).

∆ = difference between the baseline interval (1250 ms) and the stimulus percentage change. *Indicates a significant difference *p* < .05.

#### Verbal responses

The frequency of correct responses did not significantly differ across groups for the 3, 7, and 20% conditions. As expected, all participants could perceive large rhythmic fluctuations in the stimulus (Δ 20%), while only a few participants perceived the change at 3%, and roughly half of the participants perceived fluctuations at Δ 7%. It is important to note that participants were encouraged to provide their best guess if unsure; however, both answers for a given condition needed to be answered accurately to be coded as correct. Notably, only 55.6% of children with DCD provided the correct response in the isochronous condition, indicating no change in the rhythmic auditory pattern, compared to 81% of TD and 100% of TDM children (*p* < 0.023; Cramer’s V = 0.04). These results corroborate earlier findings from auditory-perceptual tasks, pointing to vulnerabilities in perceptual timing in children with DCD and enhanced beat perception among musicians (see Table 3).

**Table 3:** Percentage of correct verbal responses per condition.

	DCD	TD	TDM	*p*-value
Tapping at 0% change	55.6%	81.8%	100%	.023*
Tapping at Δ 3%	11.1%	0%	16.7%	.481
Tapping at Δ 7%	44.4%	36.4%	58.3%	.611
Tapping at Δ 20%	100%	100%	100%	–

∆ = difference between the baseline interval (1250 ms) and the stimulus percentage change.

*Indicates a significant difference between groups; *p* < .05.

### Functional connectivity patterns

Changes in functional connectivity were computed in the beta band (12–30 Hz), given its importance for sensorimotor behavior. Our initial model included all four tapping conditions and revealed main effects of Condition [*F*_(3, 63)_ = 2.82, *p* = 0.046, η_p_^2^ = 0.12] and Connectivity [*F*_(2, 42)_ = 4.82, *p* = 0.013, η_p_^2^ = 0.19]. Differences in connectivity occurred between the midline and interhemispheric networks, with increases in interhemispheric communication for musicians during small or no rhythmic fluctuations (0, Δ 3%) and during large perturbations for both the TD and TDM groups (for details, see Fig. S1 and *Differences in functional connectivity across all tapping conditions* in Supporting Information). Differences between conditions were not significant after correcting for multiple comparisons.

**Figure 2:**
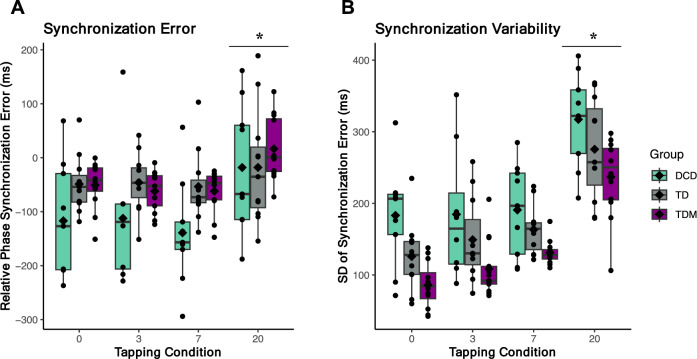
Synchronization error and variability for 0, 3, 7, and 20% conditions. (**A**) The relative phase synchronization error was calculated by taking the relative difference between the auditory cue and the finger tap. (**B**) Variability is reported as the standard deviation of synchronization error. Values closer to zero indicate less error and variability (i.e., increased synchronization accuracy). The box represents the interquartile range, the whiskers display minimum and maximum values (excluding outliers), the horizontal line represents the median, and the diamond shape indicates the mean value. Each dot represents one participant.

As synchronization strategies were the most contrasting during tapping at 0 and Δ 20%, we tested the differences between the two conditions and found main effect of Condition [*F*_(1, 21)_ = 8.66, *p* = 0.008, η_p_^2^ = 0.29] and a Group × Connectivity interaction [*F*_(4, 42)_ = 3.23, *p* = 0.021, η_p_^2^ = 0.23]. In line with the full model, TD musicians had more coherence in the interhemispheric network than midline network when tapping to a predictable beat (isochronous condition) [*t*_(8)_ = 3.24, *p* = 0.011,* d* = 1.08] (see Fig. 2). Increased interhemispheric connectivity was also present during large rhythmic fluctuations (Δ 20%) for both TD groups compared to activity in the midline network [TD; *t*_(7)_ = 4.84, *p* = 0.001,* d* = 1.71; TDM; *t*_(8)_ = 3.00, *p* = 0.017,* d* = 1.00]. Musicians further showed more interhemispheric coherence compared to midline during the Δ 20% condition [*t*_(8)_ = 2.73, *p* = 0.013,* d* = 0.91]. Therefore, when task constraints changed, shifting the synchronization strategy from predictive to reactive, differences in functional connectivity emerged for the TD and TDM groups only, while children with DCD exhibited similar connectivity patterns regardless of task difficulty (for details, see Table S2). Interestingly, children with musical training displayed more interhemispheric than intrahemispheric or midline connectivity during both predictive and reactive tapping.

## Discussion

The current study examined how small and large fluctuations in the rhythmic auditory sequence affect synchronization strategies among children with varying perceptual and motor abilities. In line with the earlier findings in adults, our results demonstrate that all children employed an anticipatory mode when making motor adjustments to smaller rhythmic fluctuations (Δ 3% and Δ 7%), even when those changes were consciously undetectable. Planned motor adjustments occurred only when participants consciously detected the larger fluctuations in the rhythmic stimuli (Δ 20%). However, at the neural level, children with DCD displayed reduced functional connectivity in the interhemispheric network compared to their TD peers with and without musical training.

### Children with DCD subconsciously employ anticipatory motor adjustments to small rhythmic fluctuations but are more variable

All groups of children were able to automatically produce motor adjustments when rhythmic perturbations were below or at the threshold of awareness. This was demonstrated by comparable phase error and variability for the Δ 3 and Δ 7% conditions. While children with DCD adopted similar anticipatory strategies (i.e., tapping ahead of the beat) as the TD and TDM groups, their synchronization error was approximately 100% larger, even when synchronizing to an isochronous sequence. This is not surprising considering that slowness of movement and increased variability are key characteristics of DCD^25^. Larger phase errors indicate that children with DCD had difficulties tracking the beat (i.e., had a larger gap between an auditory cue and a tap); nevertheless, negative mean asynchronies suggest that they could still anticipate the beat. These findings have important clinical implications as they indicate that DCD symptomatology does not affect the capacity of auditory rhythms to entrain rhythmic motor responses below levels of conscious detection, showing that rapid and temporally precise auditory computations are available in DCD despite existing perceptual-motor vulnerabilities. Moreover, the groups displayed similar inter-tap intervals, suggesting that period adaptation was unaffected in children with DCD^37^.

As expected, children started making planned adjustments only when the perturbation in the rhythmic pattern was consciously perceived at Δ 20% because this required conscious monitoring of rhythmic patterns throughout the sequence, thus shifting their tapping strategy from “predictive” to “reactive.” Importantly, although all children were aware of the Δ 20% perturbations in the stimulus, they could not identify and predict the rhythmic pattern. Contrary to our expectation, children with DCD performed similarly to other groups when large perturbations were present. This may be because the Δ 20% condition posed increased demands for all children, as evidenced by significant increases in variability across groups compared to other conditions. Indeed, previous studies have shown that differences between the DCD and TD groups become smaller when the task involves easily perceptible changes in the visual^38^ or auditory cues^29,39^. In synchronization tasks, positive error values indicate reactive strategies where participants respond to the beat (i.e., after the beat) rather than anticipate it^15^. Although the error for the Δ 20% condition was closer to zero (i.e., the beat onset) than in other conditions, it does not imply higher accuracy. Instead, it indicates that the taps were scattered around the beat onset throughout the tapping sequence (i.e., in a reactive manner), as further reflected through measures of variability. In their study with adults, Stephan and colleagues^19^ found that the error values shifted from negative to positive during large perturbations, showing reactive behavior. They also noted that participants likely implemented an automatic reactive strategy rather than a fully conscious reactive mode, given that their reaction times were shorter than typical reaction times to auditory stimuli^40^. Our findings point to a similar shift in tapping strategies, although children display considerable synchronization variability compared to adults.

### Children with DCD benefit from rhythmic entrainment despite vulnerabilities in rhythmic timing

All children were aware of large perturbations (Δ 20%), while only some participants correctly perceived small perturbations at Δ 3 and Δ 7%. Extensive literature in adults suggests that frequency fluctuations of up to 5% are perceived on a subliminal level; thus, 3 and 7% changes were below and slightly above the established perceptual threshold, respectively^23^. Considering that perceptual thresholds are higher in children^41^, it is likely that children were guessing when they were unsure. To reduce the influence of such responses, we coded their answers as correct only when both responses were correct for a given condition. Notably, the significant difference between the groups arose for the isochronous condition, where only 55% of children with DCD responded correctly. Crucially, despite having lower perceptual acuity than their peers, they were able to employ anticipatory strategies subconsciously.

As noted earlier, defining the distinction between consciously and subconsciously performed temporal adjustments of movement responses is challenging, especially since participants may or may not be aware of their adaptation strategies^19^. This challenge is exacerbated in research with children as they produce less reliable responses than older peers or adults^42^. The lower number of correct responses provided by the DCD group may be related to vulnerabilities in rhythmic timing, as evidenced by the rhythm discrimination task (see Table 1). Despite perceptual differences, children with DCD could entrain to the auditory beat and implement anticipatory responses, thus indicating that phase error adjustments are not constrained by auditory perceptual thresholds^19,22,23^. Indeed, studies in adults have demonstrated that automatic movement adjustments can be enabled through rhythmic entrainment, wherein auditory rhythms guide and organize motor output even when subtle, subliminal fluctuations in the stimuli are present^43,44^. In contrast to earlier findings by Roche and colleagues^29^, who found no significant difference in perceptual thresholds and motor adjustments between the DCD and TD groups, our findings suggest that children with DCD were able to employ anticipatory responses *despite* vulnerabilities in rhythmic timing—likely through the process of rhythmic entrainment.

### Children with DCD exhibit reduced interhemispheric connectivity during planned motor adaptations

Coherence analysis showed increases in interhemispheric communication during large perturbations in the TD and TDM groups and during small or no rhythmic fluctuations (0, Δ 3%) in musicians only, while children with DCD employed similar neural patterns regardless of task difficulty. When the two most contrasting conditions were compared (i.e., isochronous and Δ 20%), the coherence patterns further indicated the change in neural dynamics between automatic/subconscious and planned/conscious synchronization adaptation strategies. Specifically, compared to the midline network, the interhemispheric connectivity significantly increased in the TD groups as task constraints increased. Decreased activity in the midline circuitry might have occurred due to a shift from the internal timing enabled through rhythmic entrainment in the isochronous condition to consciously planned adjustments, which required increased cognitive effort. However, this shift in functional connectivity was not present for children with DCD.

To date, no studies have examined the interactions between the auditory and motor systems in children with DCD using neurophysiological or neuroimaging techniques. The existing studies using visual-motor tasks indicate that increased task constraints (e.g., speed or frequency) contribute to increased movement variability^45^ and intrahemispheric coherence for both children with DCD and their TD peers^46^. In a more complex task involving the practice of a new bimanual coordination pattern, Blais and colleagues^47^ found that increased accuracy was positively associated with intrahemispheric coherence for both the DCD and TD groups. Notably, they found that reduced interhemispheric communication correlated with decreased behavioral stability in teenagers with DCD even after practice. Several other studies suggest that the interhemispheric transfer may be altered in DCD^48,49^. While more research is critically needed, our results add to these findings, suggesting that atypical connectivity between the hemispheres may occur in children with DCD as task complexity increases. In contrast, extensive musical training has been associated with increased interhemispheric communication, modulated through the corpus callosum^50,51^. However, more research is needed to understand the role of the corpus callosum in rhythmic tasks in children with and without motor difficulties.

Despite the paucity of developmental studies, research in adults offers important insights into the mechanisms underlying motor adjustments to rhythmic auditory stimuli. For example, Tecchio et al.^20^ found that the primary auditory cortex modulated the discrimination of rhythmic fluctuations even when perturbations were not perceived consciously. Stephan and colleagues^19^ showed that the bilateral ventral mediofrontal cortex was activated when motor adjustments were performed subconsciously, while conscious recognition of rhythmic changes led to activations in the anterior cingulate and dorsolateral prefrontal cortex^19^. In the follow-up fMRI analysis, they further identified the functional cortico-cerebellar circuits that modulate different motor and cognitive aspects of rhythmic synchronization^21^, highlighting the role of the cerebellum as an optimization hub involved in not only motor control but also higher-level cognitive processes^52^. The current study extends these findings by providing a developmental perspective on conscious and subconscious movement strategies and the underlying cortical neural activity. Still, there is limited understanding of how these systems influence one another during development, especially since the neurobiological mechanisms of DCD remain unclear.

### Limitations and future directions

The cut-off point score of < 16th percentile on the MABC-2 was used to confirm the presence of motor coordination difficulties in our DCD sample. It is possible that the lower percentile (i.e., the 5th percentile, indicating more severe difficulties) would yield stronger group differences. Furthermore, two children in our sample had coexisting ADHD symptoms. Given that our sample size was modest, a separate analysis, including the DCD + ADHD subgroup, was not feasible. Considering high co-occurrence rates between DCD and ADHD^53,54^, future studies with larger samples should examine the influence of attentional resources on the shift from predictive to reactive synchronization strategies in children with coexisting DCD and ADHD.

## Conclusion

This study lays the groundwork for future research exploring the influence of awareness on rhythmic auditory-motor adjustments in children. Developmental perspectives on auditory-motor timing are critical as this process is modulated by the maturity of the underlying neural systems. Our results suggest that children with DCD may benefit from rhythmic entrainment despite the existing perceptual-motor difficulties. These insights can be utilized for the therapeutic application of auditory/rhythm-based interventions in children with DCD, as seen in adults with movement disorders.

## Methods

### Participants

Thirty-two right-handed children aged 7–11 years participated in the study (17 females, 15 males), including nine children with developmental coordination disorder (DCD), 11 typically developing children (TD), and 12 typically developing children with extensive musical training (TDM). For the musicians’ group, the requirement was to have a minimum of two years of formal piano lessons by the time of enrollment. All participants were invited from our earlier study, which involved a total of 34 children. Here, we report the results from a separate auditory-motor experiment. Children in the DCD group were screened for motor difficulties at a local hospital before their involvement in the study. They were further assessed based on DSM-5 standards^24^, including a score at or below the 16^th^ percentile on the Movement Assessment Battery for Children, 2nd Edition (MABC-2)^55^, and a parental report confirming the presence of motor difficulties in everyday situations (DCD-Q)^56^. The inclusion criteria for the TD and TDM groups included an MABC-2 score above the 30th percentile. All children scored above 70 on the cognitive tests^57^. Hand dominance was determined using the revised version of the Edinburgh Handedness Inventory^58^. For detailed information on group characteristics, see (Table 1). Additional information regarding the child’s developmental and medical history, musical training, and possible coexisting symptoms of ADHD and dyslexia were obtained through caregiver questionnaires. Children with co-occurring diagnoses and/or medical conditions affecting hearing or motor functioning (e.g., cerebral palsy, hemiplegia, or muscular dystrophy) were ineligible. The Research Ethics Board at the Holland Bloorview Kids Rehabilitation Hospital and the University of Toronto approved the study. Written informed consent was obtained from all participants and their legal guardians. The study was conducted in accordance with the Declaration of Helsinki.

### Procedures and measures

The study involved two visits. During the first visit, children completed a battery of neuropsychological and motor measures, and their caregivers reported on the child’s motor, attentional, and linguistic abilities. During the second visit, children performed the auditory-motor synchronization paradigm while their brain signals were recorded using electroencephalography (EEG). The participants also completed two auditory-perceptual tasks measuring their duration and rhythm discrimination thresholds. Participants were seated comfortably at a table in a sound-attenuated room, with two speakers positioned approximately 60 cm in front of them. The loudness of auditory stimuli was tested at a 70 dB sound pressure level and was set at a comfortable level determined by each participant. The auditory stimulus was a 1000 Hz tone for all tasks. Finger-tapping data were acquired via a Micro Light Switch by AbleNet that was placed within comfortable reach of the hand and adjusted if needed. The tasks were programmed and run on MATLAB (R2017a), and the data were stored on a computer for offline analysis.

#### Neuropsychological and auditory-perceptual measures

We conducted a battery of assessments to exclude the possibility that performance differences occurred due to cognitive and auditory-perceptual difficulties. The Kaufman Brief Intelligence Test, 2nd Edition (KBIT-2)^57^ was used to evaluate cognitive abilities, while the Digit Span Test assessed immediate auditory attention (digits forward) and working memory (digits backward)^59^. Motor abilities were tested using the MABC-2^55^ involving manual dexterity, aiming and catching, and balance, wherein lower scores implicated more severe difficulties. In addition, caregiver questionnaires inquired about the child’s possible DCD (DCD-Q)^56^, ADHD (SNAP-IV) ^60^, and dyslexia-related symptoms (the dyslexia evaluation checklist: parent form) ^61^. See *Details of neuropsychological assessments and caregiver questionnaires* in Supplementary Information.

Auditory-perceptual timing was assessed using duration discrimination (i.e., interval-based timing) and rhythm discrimination tasks (i.e., beat-based timing). Children were instructed to discriminate differences in time durations between the pairs of tones and detect deviations from the beat in the rhythmic metronome sequence. Participants completed 43 trials per task, and auditory patterns changed adaptively based on the child’s response following the 2-up-1-down staircase procedure^62^, wherein one incorrect response decreased the difficulty and two successive correct responses increased task difficulty on the subsequent trial. See *Details of auditory-perceptual tasks* in Supplementary Information.

#### Auditory-motor synchronization paradigm

**Figure 3:**
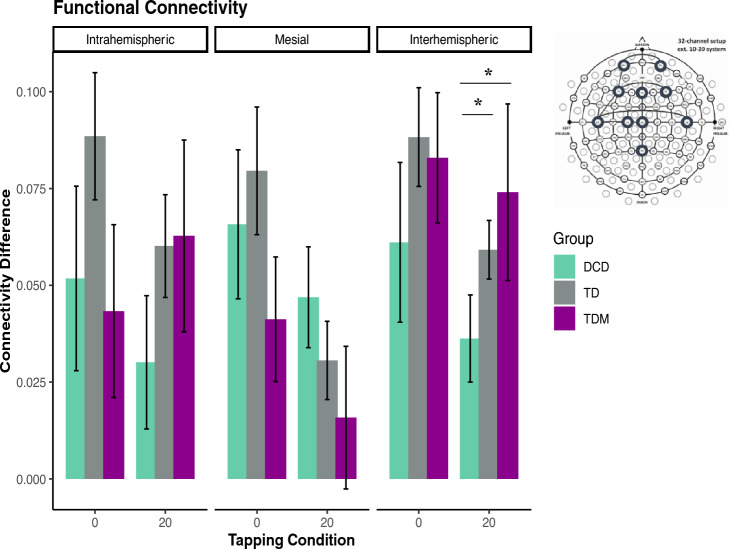
Differences in functional connectivity during synchronization tasks involving 0 and 20% perturbations in the rhythmic pattern. Differences in functional connectivity are reported for the intrahemispheric contralateral (Fp1-F3, F3-C5, C5-C1), midline/mesial (Fz-Cz, Fz-Pz), and interhemispheric networks (Fp1-Fp2, F3-F4, C5-C6) in the beta band (12–30 Hz). Error bars indicate the standard error of the mean.

The auditory-motor synchronization paradigm used here is similar to one previously tested with neurotypical adults using positron emission tomography (PET)^19,21^. Participants were instructed to synchronize their finger taps with rhythmic auditory stimuli (i.e., metronome clicks) in four different conditions: tapping to a regular rhythmic sequence (i.e., isochronous, 0% change), tapping to continuous cosine-wave modulated rhythmic sequence at 3, 7, and 20% of baseline interval of 1250 ms (Δ 3, 7, 20). Metronome clicks were generated at the mean frequency of 0.8 Hz, corresponding to the interstimulus interval (ISI) of 1250 ms. The onsets of the stimuli were systematically modulated by following a cosine-wave pattern with a period of 4 samples (see Fig. 3). The onset of every other stimulus was adjusted, alternating between early and late onsets that differed from the regular sequence by the modulation factor (example at 3% change: onsets were at 0, 1212, 2500, 3788, and 5000 ms; ISI: 1212 ms–1288 ms–1288 ms–1212 ms). This way, participants could not predict the rhythmic pattern and consequently had to adjust their taps either subconsciously or consciously. The start and the end of each tapping sequence were indicated by the high-frequency beep sound (2000 Hz). Each tapping sequence lasted 75 s, and each condition was repeated twice. Practice trials were provided to ensure participants understood the instructions. The order of the conditions was pseudo-randomized across groups and counterbalanced within groups. After completion of each tapping sequence, participants were asked to verbally report if they observed any rhythmic perturbations in the auditory sequence.

#### EEG recordings and processing

The EEG was recorded using the wireless g.Nautilus Research headset with 32 active electrodes (g.SCARABEO, g.tec medical engineering GmbH, Austria) integrated with a g.tec HIamp amplifier. The electrodes were placed according to the modified international 10–20 system, with the reference on the right earlobe and the ground electrode at AFz. The electrode impedances were kept below 50 kΩ, as recommended by the manufacturer. The data were recorded at a sampling rate of 250 Hz and transferred to the computer (MATLAB, R2017a) via Bluetooth. We pre-processed the data using the Harvard Automated Processing Pipeline for Electroencephalography (HAPPE) version 4^36^. Electrical line noise was removed at 60 Hz using the CleanLine method (Tim Mullen), and data were bandpass filtered at 0.5–80 Hz using a Hamming windowed sinc finite impulse response filter. Prior to average referencing, bad channels were interpolated, and the wavelet thresholding ICA method was used to remove ocular and muscle artifacts^36^. Participants received short training to minimize muscle artifacts and were given breaks between tasks as needed.

## Data analysis

The first ten taps of each sequence were discarded from the analysis to account for initial adjustment effects. The two sequences of each tapping condition were averaged to improve the stability of the values, resulting in approximately 100 taps in each condition. We followed the artifact removal protocols outlined in earlier sensorimotor studies^63,64^ and excluded the taps that were 50% longer or shorter than the target inter-tap intervals. We also excluded the second tap when two taps occurred within an interval of less than 100 ms. For each tapping condition, we calculated the mean inter-tap interval (ITI) as a measure of tapping consistency (i.e., tapping rate) and the relative phase synchronization error as a measure of synchronization accuracy by taking the relative difference between the metronome click and the finger tap. Negative mean values indicated that the taps preceded the auditory beat, and positive mean values suggested that the taps occurred after the beat. In addition, the standard deviation for both the ITI and SE was computed as a measure of tapping and synchronization variability, respectively. For each sequence, participants provided verbal responses indicating the presence or absence of fluctuations in the stimulus (e.g., “yes” indicated that they perceived the change in the rhythmic cue). Each condition was repeated twice. Both answers in each condition needed to be answered accurately to be coded as correct. If participants reported being fatigued, the session ended after the first run.

### EEG data analysis

The MNE package (MEG + EEG Analysis and Visualization) was used for the EEG analyses. We performed coherence analysis between each channel pair, ${C}_{xy}$, based on time-resolved estimates of cross-spectral densities (${S}_{xy}$), and power spectral densities (${S}_{xx}$, ${S}_{yy}$)^65^.

$${C}_{xy}=\frac{|E\left[{S}_{xy}\right]|}{\sqrt{E\left[{S}_{xx}\right]\times E[{S}_{yy}]}}$$

The data were segmented into 600 ms epochs (−100 to 500 ms) around the tap onset. As the data were segmented to movement response, the number of EEG epochs corresponded with the number of taps obtained in each condition. Each condition (Isochronous, 3, 7, and 20% change) was repeated twice, and each tapping sequence consisted of 60 auditory stimuli (i.e., 60 expected taps). This resulted in a total of 100 epochs per condition for each participant after the first ten taps of each tapping sequence were discarded from the analysis to account for initial adjustment effects. Baseline correction was applied by subtracting the average of the pre-movement onset between −500 and −300 ms. Coherence was computed in the beta band (12–30 Hz) using Morlet wavelets with one cycle. The spectral connectivity was estimated over time as the coherence between channel pairs. All channels were included except for the PO7, PO3, PO4, PO8, and Oz electrodes. The selected electrodes in the fronto-central network were grouped as follows: intrahemispheric contralateral (Fp1-F3, F3-C5, C5-C1), midline (Fz-Cz, Fz-Pz), and interhemispheric (Fp1-Fp2, F3-F4, C5-C6). Before each tapping sequence, the resting state EEG data were recorded for 30 s and were later segmented into non-overlapping epochs of the same length as the task epochs. The resting state coherence was subtracted from the tapping coherence for each condition^66^. The matrix-style difference plots were generated by calculating the difference between the mean task coherence and mean resting state coherence at each link. To assess the differences in distribution between task and resting state coherence, the two-sample Kolmogorov–Smirnov (KS) test was applied across epochs. If the task and resting state coherence distributions did not statistically differ at *p* < 0.01, the coherence differences were set to zero. The KS test was applied over epochs for each individual with the *p*-value threshold of < 0.01. The resulting masked differences were then averaged across participants within each group. Finally, the coherence values of the selected links within each grouping were averaged before statistical analysis.

### Statistical analyses

Statistical analyses were performed using R Studio (version 4.3.0). The significance threshold for all data analyses was set at *p* < 0.05. We assessed data normality using the Shapiro–Wilk test and the homogeneity of variances assumption using Levene’s test. When the assumptions were violated for at least two groups (*p* < 0.05), we reported the Kruskal–Wallis H and Welch’s test, respectively. Chi-square tests and one-way analyses of variance (ANOVAs) were used to assess group differences across demographic variables and auditory-perceptual tasks and the distribution of participants’ verbal responses. Fisher’s exact test values were reported when any expected cell count in the contingency table was less than five. For the effect size measures, we calculated η_p_^2^ for the ANOVAs and Cramer’s V for nominal categorical variables. Mixed-design ANOVAs involved Condition (4 or 2 levels) and Connectivity (3) as the within-subjects factors and Group (3) as the between-subjects factor. We used the *lmer* function (*lme4* package) in R to fit the ANOVA models. Additional analyses of covariance (ANCOVAs) were performed for tapping data with age as a covariate. The assumption of sphericity was assessed using Mauchly’s test, and the Greenhouse–Geisser correction was applied to the degrees of freedom when appropriate. For significant effects, post-hoc paired and independent samples *t*-tests tests were computed using the Bonferroni correction.

### Ethics approval

This research was reviewed and approved by the Holland Bloorview Kids Rehabilitation Hospital and the University of Toronto Research Ethics Board.

### Supplementary Information

Supplementary Information.

## Data Availability

The datasets generated and/or analyzed during the current study are available from the corresponding author upon reasonable request.
