# Supplementary material for: Children with developmental coordination disorder display atypical interhemispheric connectivity during conscious and subconscious rhythmic auditory-motor synchronization
Source: Sci Rep. 2024 Aug 28;14:19954. doi: 10.1038/s41598-024-69807-4 (PMC11358286; doi:10.1038/s41598-024-69807-4)
Supplement: Supplementary file 1 — Supplementary Information. [file 41598_2024_69807_MOESM1_ESM.docx]

**SUPPLEMENTARY INFORMATION**

**Children with developmental coordination disorder display atypical interhemispheric connectivity during conscious and subconscious rhythmic auditory-motor synchronization**

Marija Pranjić^1,2*^, Jason Leung^2^, Ka Lun Tam^2^, Helene Polatajko^3^, Timothy Welsh^4^, Tom Chau^2,5†^, & Michael Thaut^1,6†^

† Senior authors

^1^ Music and Health Research Collaboratory, Faculty of Music, University of Toronto, Canada

^2^ Bloorview Research Institute, Holland Bloorview Kids Rehabilitation Hospital, Canada

^3^ Department of Occupational Science and Occupational Therapy, Rehabilitation Sciences Institute, Faculty of Medicine, University of Toronto, Canada

^4^ Centre for Motor Control, Faculty of Kinesiology & Physical Education, University of Toronto, Canada

^5^ Institute of Biomedical Engineering, University of Toronto, Canada

^6^ Institute of Medical Science and Rehabilitation Research Institute, Faculty of Medicine, University of Toronto, Canada

*Corresponding Author: Marija Pranjić, marija.pranjic@mail.utoronto.ca

*Details of neuropsychological assessments and caregiver questionnaires*

The Kaufman Brief Intelligence Test, 2nd Edition (KBIT-2) [1] is a standardized assessment tool for assessing verbal and nonverbal intelligence in individuals aged 4 to 90. Raw values were converted into standard scores, and a score above 70 indicated normal cognitive functioning. We used the Digit Span Test to evaluate immediate auditory attention (Digits Forward) and working memory (Digits Backward) [2]. Participants first repeated increasing spans of digits in the same order as they were presented (Digit Span Forward) and then repeated the procedure in a reversed order (Digits Backward) until two consecutive incorrect responses were provided within a specific span length. The final score reflected the sum of correctly recalled trials for each task. Motor coordination abilities were assessed using the Movement Assessment Battery for Children, 2nd Edition (MABC-2) [3], which includes a variety of tasks testing manual dexterity, aiming and catching, and balance. Standardized scores are transformed into percentiles, and a score at or below the 16^th^ percentile indicates motor coordination difficulties. Additionally, caregivers completed three questionnaires to better characterize their child’s abilities across motor, attention, and speech and language domains.

The Developmental Coordination Disorder Questionnaire (DCD-Q) [4] is a parent-based screening tool for identifying children at risk for DCD. Caregivers are asked to compare their child’s motor performance to that of their peers using a 5-point Likert scale. The Swanson, Nolan, and Pelham questionnaire (SNAP-IV) is commonly used for identifying attention difficulties in children from a parent or teacher perceptive [5]. Using a 0 to 3 rating scale, caregivers rated their child’s symptoms of inattention, hyperactivity/impulsivity, and oppositional defiant disorder. Scores above 27 for inattention and hyperactivity/impulsivity and above 24 for oppositional defiant disorder are considered clinically significant. Language development was assessed using the Dyslexia Evaluation Checklist: Parent Form (Woodcock-Johnson IV) [6], which included questions regarding the child’s reading development, prereading and reading skills, and spelling and writing abilities.

*Details of auditory-perceptual tasks*

Duration and rhythm discrimination tasks were used to assess potential differences in perceptual timing. The duration task consisted of changes in tone durations in the absence of an underlying beat, while the rhythm task involved deviations from the beat in the rhythmic metronome sequence. Participants verbally reported whether they perceived a change in duration or rhythmic pattern after each trial (i.e., an adaptive two-alternative forced-choice paradigm [7], and the experimenter recorded the response by pressing the corresponding key. This way, we aimed to minimize the influence of motor skill differences between groups.

In the duration discrimination task, there were two tones in each trial separated by 1,120 ms (tone frequency = 1 Hz). The first tone (i.e., the standard tone) lasted 500 ms while the following tone (i.e., the target tone) changed adaptively, varying in duration between 260 and 500 ms in 15 ms step sizes. The change was determined by the 2-up-1-down staircase paradigm [8], and participants had to judge if the duration of the two tones was the same or different.

The rhythm discrimination task involved two 5-tone sequences per trial separated by 1,120 ms (tone duration = 80 ms). Both sequences had a constant inter-onset interval (IOI) of 500 ms, except for the last IOI in the second sequence, which was always shorter (ranging between 335 and 500 ms, with a step size of 15 ms). Children were asked to determine whether the two rhythmic sequences were the same or different. Both tasks included five practice trials with feedback, wherein the target tone/sequence was fixed at 250 ms (easy discrimination level). Five probe trials were randomly placed within each task and were fixed at 250 ms (the same as practice trials). Participants needed to respond correctly to at least 4 of the 5 probe trials. The probe trials were not included in the analysis but were used to indicate whether children remained attentive throughout the experiment.

Table S1. Participant characteristics for the subgroup included in the EEG analysis.

|  | DCD | TD | TDM | *p*-value |
| --- | --- | --- | --- | --- |
| *N* | 7 | 8 | 9 | - |
| Age (months) | 107.57± 17.96 | 105.12 ± 12.65 | 107.11 ± 16.68 | .949 |
| Non-Caucasian | 33.33 % | 25 % | 87.5 % | .002* |
| Income < $100,000 | 33.33 % | 40 % | 11.1 % | .301 |
| Laterality Quotient (RH) | 92.86 % | 100 % | 100 % | .31 |
| KBIT-2 Composite IQ | 110.43 ± 22.99 | 118.88 ± 9.48 | 120.44 ± 10.03 | .386 |
| DST Forward | 8 .14 ± 1.35 | 8 ± 1.85 | 8.33 ± 1.41 | .907 |
| DST Backward | 4 ± 2.24 | 5.12 ± 2.9 | 5.78 ± 1.48 | .311 |
| MABC-2 Percentile | 7.29 ± 4.57 | 73.25 ± 11.77 | 60.78 ± 13.87 | < .001* |
| DCD-Q | 29.43 ± 9.81 | 67.38 ± 7.11 | 69.44 ± 5.48 | < .001* |
| Duration Discrimination | 36.45 ± 4.51 | 34.91 ± 5.79 | 31.33 ± 2.79 | .082 |
| Rhythm Discrimination | 22.62 ± 7.14 | 20.07 ± 3.17 | 14.47 ± 1.23 | < .003* |

*Note.* DCD = developmental coordination disorder; TD = typically developing children; TDM = typically developing musicians; Laterality Quotient (RH) = right-handedness measures by Edinburgh Handedness Inventory; KBIT-2 = The Kaufman Brief Intelligence Test, 2nd Edition; MABC-2 = The Movement Assessment Battery for Children, 2nd Edition; DST = The Digit Span Test; DCD-Q = The Developmental Coordination Disorder Parent Questionnaire. Difference tests were one-way ANOVAs (reported as *M* ± *SD*) and chi-square tests for categorical variables. * Indicates a significant difference between groups; *p* < .05.

*Differences in functional connectivity across all tapping conditions*

When all four tapping conditions were included in the model (3×4×3 ANOVA), changes in connectivity patterns occurred between the midline and interhemispheric networks, wherein coherence in the interhemispheric network increased for musicians (TDM) in an isochronous condition [*t*_(9)_ = 3.24, *p* = .012, *d* = 1.08] and during small rhythmic fluctuations (Δ 3) [*t*_(9)_ = 9.88, *p* < .001, *d* = 3.29]. During large perturbations, both the TD [*t*_(8)_ = 4.84, *p* = .002, *d* = 1.71] and TDM groups [*t*_(9)_ = 3.00, *p* = .017, *d* = 1.00] displayed increased communication between the hemispheres compared to activity in the midline network. Connectivity differences are illustrated in Fig. S1.

Table S2. Mean functional connectivity values across grouping networks.

|  | DCD  (*n* = 7) | TD  (*n* = 8) | TDM  (*n* = 9) |
| --- | --- | --- | --- |
| Intrahemispheric | | | |
| Isochronous  Δ 3%  Δ 7%  Δ 20% | .052 ± .063  .075 ± .043  .041 ± .046  .030 ± .046 | .088 ± .046  .083 ± .066  .079 ± .051  .060 ± .038 | .043 ± .067  .037 ± .052  .054 ± .050  .063 ± .074 |
| Midline | | | |
| Isochronous  Δ 3%  Δ 7%  Δ 20% | .066 ± .051  .074 ± .071  .042 ± .048  .047 ± .034 | .080 ± .047  .068 ± .028  .071 ± .051  .031 ± .029 | .041 ± .048  .035 ± .034  .047 ± .050  .016 ± .055 |
| Interhemispheric |  |  |  |
| Isochronous  Δ 3%  Δ 7%  Δ 20% | .061 ± .055  .060 ± .044  .057 ± .026  .062 ± .027 | .088 ± .036  .095 ± .037  .083 ± .041  .062 ± .034 | .083 ± .051  .081 ± .030  .059 ± .055  .062 ± .055 |

*Note.* Reported as M ± SD; M = mean; SD = standard deviation.

**
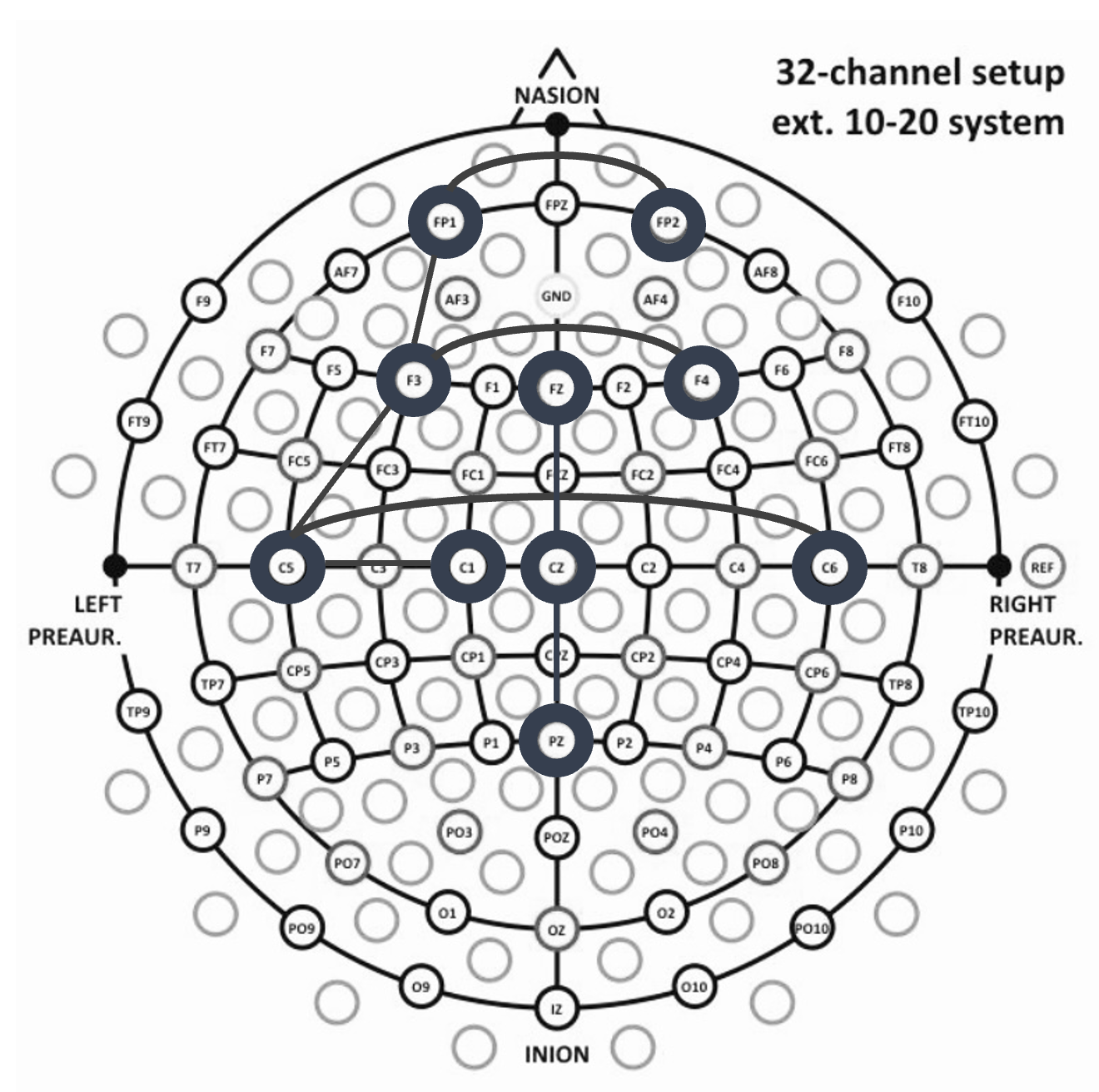
**

Figure S1. Differences in functional connectivity across all tapping conditions are reported for the intrahemispheric contralateral (Fp1-F3, F3-C5, C5-C1), midline/mesial (Fz-Cz, Fz-Pz), and interhemispheric networks (Fp1-Fp2, F3-F4, C5-C6) in the beta band. Error bars indicate the standard error of the mean.

**References**

1. Kaufman, A., & Kaufman, N. (2004). *Kaufman brief intelligence test* (2nd ed.). Circle Pines, MN: AGS.

2. Turner, M. & Ridsdale, J. The Digit Memory Test. *Dyslexia Action* (2004).

3. Henderson, S. E. & Barnett, A. L. *Movement assessment battery for children.* London: Psychological Corporation (1992).

4. Wilson, B. N. *et al.* Psychometric Properties of the Revised Developmental Coordination Disorder Questionnaire. *Phys. Occup. Ther. Pediatr.* **29**, 182–202 (2009).

5. Swanson, J. M. *et al.* Categorical and dimensional definitions and evaluations of symptoms of ADHD: history of the SNAP and the SWAN rating scales. *Int. J. Educ. Psychol. Assess.* **10**, 51-70 (2012).

6. Proctor, C. M., Mather, N., Stephens-Pisecco, T. L., & Jaffe, L. E. Assessment of Dyslexia. *Communique* **46**, 1-20 (2017).

7. Kingdom, F. A. A., & Prins, N. *Psychophysics: A Practical Introduction.* (Cambridge, MA: Academic Press., 2010).

8. Levitt, H. C. C. H. Transformed up‐down methods in psychoacoustics. *J. Acoust. Soc. Am.* **49,** 467-477 (1971).
